# Exploring the Feasibility of Service Integration in a Low-Income Setting: A Mixed Methods Investigation into Different Models of Reproductive Health and HIV Care in Swaziland

**DOI:** 10.1371/journal.pone.0126144

**Published:** 2015-05-15

**Authors:** Kathryn Church, Alison Wringe, Simon Lewin, George B. Ploubidis, Phelele Fakudze, Integra Initiative, Susannah H. Mayhew

**Affiliations:** 1 Department of Population Health, London School of Hygiene & Tropical Medicine, London, United Kingdom; 2 Global Health Unit, Norwegian Knowledge Centre for the Health Services, Oslo, Norway; 3 Health Systems Research Unit, Medical Research Council of South Africa, Cape Town, South Africa; 4 Centre for Longitudinal Studies, Department of Quantitative Social Science, Institute of Education, University of London, London, United Kingdom; 5 PSI, Mbabane, Swaziland; 6 Department of Global Health and Development, London School of Hygiene & Tropical Medicine, London, United Kingdom; Brighton and Sussex Medical School, UNITED KINGDOM

## Abstract

Integrating reproductive health (RH) with HIV care is a policy priority in high HIV prevalence settings, despite doubts surrounding its feasibility and varying evidence of effects on health outcomes. The process and outcomes of integrated RH-HIV care were investigated in Swaziland, through a comparative case study of four service models, ranging from fully integrated to fully stand-alone HIV services, selected purposively within one town. A client exit survey (n=602) measured integrated care received and unmet family planning (FP) needs. Descriptive statistics were used to assess the degree of integration per clinic and client demand for services. Logistic regression modelling was used to test the hypothesis that clients at more integrated sites had lower unmet FP needs than clients in a stand-alone site. Qualitative methods included in-depth interviews with clients and providers to explore contextual factors influencing the feasibility of integrated RH-HIV care delivery; data were analysed thematically, combining deductive and inductive approaches. Results demonstrated that clinic models were not as integrated in practice as had been claimed. Fragmentation of HIV care was common. Services accessed per provider were no higher at the more integrated clinics compared to stand-alone models (p>0.05), despite reported demand. While women at more integrated sites received more FP and pregnancy counselling than stand-alone models, they received condoms (a method of choice) less often, and there was no statistical evidence of difference in unmet FP needs by model of care. Multiple contextual factors influenced integration practices, including provider de-skilling within sub-specialist roles; norms of task-oriented routinised HIV care; perceptions of heavy client loads; imbalanced client-provider interactions hindering articulation of RH needs; and provider motivation challenges. Thus, despite institutional support, factors related to the social context of care inhibited provision of fully integrated RH-HIV services in these clinics. Programmes should move beyond simplistic training and equipment provision if integrated care interventions are to be sustained.

## Introduction

Promoting integrated health care is a common public health priority in settings dominated by ‘vertical’ health programmes. This is particularly true in high-HIV prevalence settings in sub-Saharan Africa, where the impetus to rapidly scale-up access to HIV care and treatment (HCTx) in the early 2000s led to the predominance of vertical programmes and stand-alone HIV services in many settings [1]. While rapid service proliferation was successful at getting many people living with HIV (PLHIV) onto anti-retroviral therapy (ART), concerns emerged about the sustainability of such an approach, the potential duplication of effort and services, and the impact of mass investment in one disease on the broader health system [2].

Integration of HIV with other health services was seen as an important mechanism to overcome verticalisation and strengthen health systems [3], and also formed an important component of ART decentralisation policies [4]. The success of ART and the transformation of HIV into a chronic condition also implied the need to tackle the multiplicity of clinical and psycho-social needs of PLHIV [5]. Addressing their distinct reproductive health (RH) needs has been highlighted as a particular concern by leaders of the HIV and RH communities [6–7]. RH also plays a critical HIV prevention role, through reduction of unintended pregnancies (thus impacting on prevention of mother-to-child transmission) [8], and through the promotion of condom use and sexual behaviour counselling to prevent onwards transmission [9]. The longitudinal patient contact of HCTx also offers synergies to address RH needs, which require repeated contacts with health services [10].

A growing body of literature, however, indicates that the policy rhetoric of integration may be at odds with the complex service delivery reality in lower-income settings [11]. Challenges include the capacities and willingness of providers to deliver a broader package of care, in particular when extra demands are made without concomitant improvements in infrastructure, working conditions, or salary [12–14]; relationships between clients and providers, and challenges in straying from practice routines that lie at the heart of traditional medical culture [15]; relations among providers, including professional territorialism and lack of role definition [16–17]; and various health systems barriers, including infrastructure, equipment, data management, managerial and human resource factors [14, 18–19]. In HIV clinics, burgeoning client populations can result in cutbacks of non-core ART services [20], despite complex client needs.

Conceptually, there is little agreement on what ‘integrated care’ actually means, and even less on how it is measured [21]. It can imply the amalgamation of two previously separate components of care, or the addition of a new intervention into an existing service [22]. In this paper we consider the provision of RH (including FP, pregnancy counselling, and condom promotion) with HCTx services. We investigated the process and outcomes of RH-HIV integration in Swaziland, the country with the world’s highest HIV prevalence (26% among adults) [23], and where addressing the RH needs of PLHIV and the decentralisation of HCTx into generalist health services were policy priorities [24]. A comparative case study design of four different models of HCTx was used, allowing investigation of service structure within its real-life context and cross-case analysis [25]. The study formed a sub-component of a multi-country investigation of RH-HIV service integration in Africa, the Integra Initiative [26]. The aims of this paper are (i) to describe how RH care was being delivered within the four service models and the extent to which it was as integrated as it ‘should’ have been (i.e. according to *a priori* clinic labelling); (ii) to investigate whether integrated RH-HIV models were more effective in addressing FP needs than stand-alone models; and (iii) to qualitatively explore the factors affecting the delivery of integrated care in this context.

## Materials and Methods

### Study setting

The four case study clinics were identified purposively within Swaziland’s largest town, Manzini, and were all accessible to the same geographic catchment population. Each clinic was identified by facility managers and national programme managers as representing a specific model of integrated or stand-alone HIV service delivery. They were the only HCTx facilities operating in Manzini at the time of the study and all reported offering free ART services. ART services were provided by a mix of doctors and nurses, with former usually initiating patients, and nurses providing monitoring. Details on the four clinics have been published previously [27–28], but in summary the reported models were:

#### Clinic A (fully integrated)

RH-HIV service with all services theoretically available from one provider in one room (NGO-run; client load of 6 patients/ART provider/day)

#### Clinic B (partially integrated)

primary care clinic, with different RH and HIV service components offered by different providers in separate rooms within one building (Government-run, supported by NGOs; client load of 13.5 patients/ART provider/day)

#### Clinic C (partially stand-alone)

HIV unit operating on the campus of the district hospital in separate building (Government-run, supported by NGOs; client load of 24 patients/ART provider/day)

#### Clinic D (fully stand-alone)

HIV clinic (NGO-run; client load of 15.8 patients/ART provider/day)

### Quantitative methods

An exit survey among HIV care clients (N = 602) (either pre-ART or on ART), aged ≥18, was conducted in 2009. The survey methods have been described in detail elsewhere [27–28]. Clients were selected for interview using a systematic random sampling technique. A sample of 200 per clinic was required to detect a difference of 15% in unmet FP needs between sites (α 0.05, power 80%), anticipating that 70% of the clinic population was female. Sample size at Clinic A was anticipated to be lower due to low client load (ART had only been operational for 8 months prior to the survey), and thus attempts were made to interview all clients attending services during the survey period, rather than a probability sample. Questions covered socio-demographic and health characteristics, services received or referred for, providers seen, services desired, and RH services accessed. Service contacts for FP were measured in two ways. Firstly, since all clients were interviewed following an HIV service contact, use of component HIV and SRH sub-services was documented, covering HIV care (ART refill, pre-ART, weighing/blood pressure check, group counselling, ART initiation, ART side-effects consultation, consultation for HIV-related problems, HIV general consultation, CD4 testing, blood tests) and SRH care (condom provision, screening for sexually transmitted infections, ante-natal care, preventing mother to child transmission service, FP, pap smear or post-natal care). Secondly, RH services used since testing HIV positive were recorded, including advice on FP methods, provision of FP methods, advice on getting pregnant, counselling on condom use, and provision of condoms. Unmet FP needs were measured among sexually active fecund women of reproductive age (18–49), and were defined as not wanting another child in the next 2 years and not currently using a modern contraceptive, or having a current mistimed or unwanted current pregnancy. Given high rates of reported condom use in this population (80% of modern method users [29]), contraceptive protection was considered achieved only if condom users reported consistent use.

Statistical analysis was conducted using STATA 12.0. Differences in the mean number of services used and providers seen across sites were tested with the analysis of variance (ANOVA) test, and between sites using the Tukey-Kramer pairwise comparison. Differences in the proportions of clients at each site receiving RH services were tested using χ2 tests. Logistic regression was used to test the hypothesis that unmet FP needs were higher at more integrated sites compared to fully stand-alone Clinic D. Potential confounders were identified based on a literature review and formative research, and covered socio-economic, geographic, SRH and other health-related variables (see Supporting Information S1 Fig). A crude analysis examined the associations between potential confounders with the exposure (clinic model) and with the outcome (unmet FP needs), using χ2 tests. Stratum-specific odds ratios of the association between clinic model and unmet FP needs were tabulated across potential effect modifiers (identified *a priori*), using the Mantel- Haenszel method: no significant interaction was identified. Since the aim of the analysis was to adjust for baseline differences between clinics, a multivariable model was constructed with all conceptually related variables retained in the model.

### Qualitative methods

In-depth interviews (IDIs) were conducted with 16 health care providers (5 doctors, 11 nurses); and with 22 HIV clients across the four sites. Sampling for both was purposive. All providers delivering HCTx services were invited to interview and none refused; some also offered SRH services (Table 1). At Clinic C, a larger site, five out of seven were interviewed based on availability. Details on the client sample have been published before [27], but in summary, 5–6 were interviewed at each site, with a mean age of 31 (range 22–45). Clients were initially interviewed at the clinic on the day of their ART initiation, and then again 2 and 6 months later, in order to investigate how RH was addressed in those first months of contact. The aim was to interview at least two men and one pregnant woman per clinic, but potentially more if data saturation was not achieved. Clients were invited during group counselling, and were interviewed in a private room or area of the clinic, initially. Follow-up interviews were conducted at a town meeting room or at home. Client interviews were conducted in SiSwati, provider interviews in English.

**Table 1:** Characteristics of provider IDI sample.

Characteristic	Category	No. respondents (N = 16)
Clinic	Clinic A	4
	Clinic B	4
	Clinic C	5
	Clinic D	3
Mean age (range)	36 years (range 26–50 years)	
Profession	Doctors	5
	Nurses	11
Work focus (observed)	ART providers	13
	SRH-HIV nurses	3
Role/specialisation (self-reported)	Generalists (only)	10
	Generalist/ART specialist	3
	ART /nurse anaesthetist	1
	ART specialist	1
	ART and medical/surgical nursing	1
Mean years working in clinic	4.5 yrs	
Recent training (in last 3 years)	ART	13
	PMTCT	12
	FP	6
	FP for PLWH	8
	STI	7
	Cervical cancer screening	8

Interviews were recorded, transcribed, and translated into English (when needed). Data were analyzed through an iterative process, including stages of (i) data familiarization, through transcript review; (ii) development of coding framework (using NVivo 8.0), derived deductively from the research questions and inductively from the data, with a sub-sample of 3 client and 3 provider transcripts double coded by two researchers; (iii) abstraction of coded data into thematic matrices, allowing a constant comparative approach [30] across clinics and cases (each matrix addressed an over-arching study theme, and findings here are derived from the matrix on ‘context’); and (iv) interpretation, methodological synthesis and write-up. Results are organised into a broad framework informed by a critical realist perspective [31], which groups contextual factors influencing policy implementation into four areas: individual, inter-personal, infrastructural/systems, and institutional factors.

### Ethics

Ethical approval for the study was obtained from the Ethical Committee at the London School of Hygiene & Tropical Medicine (approval no. 5436) and from the Swaziland Scientific and Ethics Committee (approval no. MH/139). Written informed consent was obtained from all participants, prior to interview. Consent forms were signed and stored in locked cabinets by the research team, a procedure approved by the relevant ethical committees.

## Quantitative Findings: How Integrated Was Care?

A detailed description of the survey population has been published elsewhere [27–28, 31][27,28,31][27,28,31]. In summary, most respondents were female (79%), the largest groups were in their 30s (37%) and fell into the lowest income bracket (34%); a majority had achieved some secondary education (59%); most were on ARVs (82%); and the largest group had been enrolled on ART for between 6 months and 2 years (44%).

Table 2 summarises data on services accessed on the day of survey and RH services accessed since testing HIV positive. HIV visits were fragmented across different types of providers across all sites, usually for sub-components of HIV care itself (e.g. weighing/blood pressure check, ART refill consultation, drug dispensing), with clients seeing on average 2.3 providers per visit for 2.8 sub-services. Among the 1656 provider contacts, only 73 (4%) were for RH services, and even fewer, 6 (0.4%), were for condom provision or FP services (see S1 Table). While the number of provider contacts was lowest at partially integrated Clinic B, the number of sub-services obtained per provider at the most “integrated” Clinic A (1.3, SD 0.4), was also low and not different statistically to any other site (p>0.05, Tukey-Kramer pairwise comparisons). Service access was also not compensated for by referral, which was very limited (Table 3): only 42 clients were referred overall (the majority to the laboratory at the hospital), and only three of these were for RH services.

**Table 2:** Providers and services accessed on day of survey and since testing HIV positive, by clinic.

	Clinic A		Clinic B		Clinic C		Clinic D		All clinics		
	(Fully integrated)		(Partially integrated)		(Partially stand-alone)		(Fully stand-alone)				
RH/HIV service use on day of survey (mean, (SD)):											P value (F stat)
Mean no. providers seen during visit	2.5	(1.0)	1.8	(0.8)	2.8	(0.8)	2.2	(0.4)	2.3	(0.8)	58.79 (<0.001)
Mean no. RH and HIV sub-services accessed	3.1	(1.5)	2.3	(1.1)	3.1	(1.0)	2.7	(0.9)	2.8	(1.1)	17.28 (<0.001)
Mean no. sub-serivces per provider contact	1.3	(0.4)	1.4	(0.6)	1.1	(0.3)	1.2	(0.3)	1.2	(0.4)	10.35 (<0.001)
Mean no. additional RH or HIV services desired	2.7	(2.1)	4	(2.8)	3.2	(2.2)	2.7	(1.9)	3.2	(2.4)	10.08 (<0.001)*
**SRH services received since testing HIV positive (among women, N = 475) (%(N)):**											**(χ2)**
FP advice	55.6	(30)	73.4	(105)	60.2	(80)	40.7	(59)	57.6	(274)	<0.001
FP method provision	42.6	(23)	38.5	(55)	19.6	(26)	39.3	(57)	33.8	(161)	0.001
Pap smear	35.2	(19)	10.5	(15)	13.5	(18)	10.3	(15)	14.1	(67)	<0.001
Advice on getting pregnant	59.3	(32)	69.2	(99)	70.7	(94)	48.3	(70)	62.0	(295)	<0.001
**SRH services received since testing HIV positive (all clients, N = 602) (%(N)):**											**(χ2)**
Counseling on condom use	76.1	(54)	77.9	(127)	94.9	(169)	86.8	(165)	85.4	(515)	<0.001
Provision of condoms	40.9	(29)	38.7	(63)	55.6	(99)	77.4	(147)	56.1	(338)	<0.001
Advice on sexual health	15.5	(11)	12.3	(20)	15.7	(28)	10.5	(20)	13.1	(79)	0.451
Sexual health screening	45.1	(32)	57.1	(93)	75.8	(135)	41.6	(79)	56.2	(339)	<0.001
**Total no. clients:**		**71**		**163**		**178**		**190**		**602**	

**Table 3:** Referrals, by clinic.

Variable	Clinic A		Clinic B		Clinic C		Clinic D		All clinics		P value
	(Fully integrated)		(Partially integrated)		(Partially stand-alone)		(Fully stand-alone)				
	%	(N)	%	(N)	%	(N)	%	(N)	%	(N)	χ2
**Clients referred outside building**											
No	91.6	(65)	93.9	(153)	86.5	(154)	99.0	(188)	93.0	(560)	<0.001
Yes	8.5	(6)	6.1	(10)	13.5	(24)	1.1	(2)	7.0	(42)	
Total (all clients):*	**100.0**	**(71)**	**100.0**	**(163)**	**100.0**	**(168)**	**100.0**	**(190)**	**100.0**	**(602)**	
**Among those referred, service referred for:**											
Lab	55.6	(5)	20.0	(2)	60.0	(15)	66.7	(2)	51.1	(24)	
Pharmacy	11.1	(1)	30.0	(3)	36.0	(9)	0.0	(0)	27.7	(13)	
STI	11.1	(1)	20.0	(2)	4.0	(1)	33.3	(1)	10.6	(5)	
PNC	0.0	(0)	20.0	(2)	0.0	(0)	0.0	(0)	4.3	(2)	
Cardiology	11.1	(1)	0.0	(0)	0.0	(0)	0.0	(0)	2.1	(1)	
FP	11.1	(1)	0.0	(0)	0.0	(0)	0.0	(0)	2.1	(1)	
*Missing*	*0*.*0*	*(0)*	*10*.*0*	*(1)*	*0*.*0*	*(0)*	*0*.*0*	*(0)*	*2*.*1*	*(1)*	
**Total referred (N = 42 cases, 47 referral services)**	**100.0**	**(9)**	**100.0**	**(10)**	**100.0**	**(25)**	**100.0**	**(3)**	**100.0**	**(47)**	

*1 respondent had missing data on referral

A lack of integrated service delivery did not stem from low client demand: clients desired an average of 3.2 additional services each, ranging from 2.7 services at Clinics A and D to 4.0 at Clinic B (Table 4). More information was desired on various health topics, including FP services (36%), and counselling on sexual functioning/libido (31%).

**Table 4:** Desired additional services on day of survey, by clinic.

Variable	Clinic A		Clinic B		Clinic C		Clinic D		All clinics		P value
	(Fully integrated)		(Partially integrated)		(Partially stand-alone)		(Fully stand-alone)				
**Additional services desired (mean, SD)**	2.7	(2.1)	4.0	(2.8)	3.2	(2.2)	2.7	(1.9)	3.2	(2.4)	10.08* (<0.001)
**Clients who would have liked to receive (%(N)):**											
No more services	15.5	(11)	11.0	(18)	11.2	(20)	19.0	(36)	14.1	(85)	<0.001
1–2 additional services	32.4	(23)	24.5	(40)	32.0	(57)	23.2	(44)	27.2	(164)	
3–4 additional services	31.0	(22)	23.9	(39)	32.0	(57)	41.6	(79)	32.7	(197)	
≥ 5 additional services	21.1	(15)	40.5	(66)	24.7	(44)	16.3	(31)	25.9	(156)	
**Clients who would have liked to receive (%(N)):**											
TB services	50.7	(36)	63.8	(104)	74.7	(133)	76.3	(145)	69.5	(419)	<0.001
Info on ART	60.6	(43)	68.1	(111)	63.5	(113)	56.8	(108)	62.4	(376)	0.163
STI services	43.7	(31)	47.9	(78)	59.0	(105)	40.5	(77)	48.4	(292)	0.004
FP services	32.4	(23)	44.2	(72)	46.6	(83)	19.0	(36)	35.7	(215)	<0.001
Counselling on sexual functioning	19.7	(14)	42.3	(69)	20.2	(36)	36.8	(70)	31.3	(189)	<0.001
Counselling on how/when to get pregnant	36.6	(26)	46.6	(76)	26.4	(47)	14.2	(27)	29.4	(177)	<0.001
Child health services	16.9	(12)	43.6	(71)	15.7	(28)	13.7	(26)	22.9	(138)	<0.001
Advice on pregnancy/childbirth	11.3	(8)	30.7	(50)	11.8	(21)	10.0	(19)	16.4	(99)	<0.001
Pregnancy testing	2.8	(2)	11.0	(18)	5.6	(10)	6.3	(12)	7.0	(42)	0.087
**Total no. clients**		**71**		**163**		**178**		**190**		**602**	

*Anova F Statistic (p value)

Regarding RH services accessed since testing HIV positive, while most (85%) received counselling on condom use, other services were less consistently accessed across all models (Table 2). Among women, 62% received advice on getting pregnant, 58% on FP, and 34% received FP methods. 56% of all clients (male and female) were provided condoms. These indicators did vary by clinic but integrated sites were not consistently better than Clinic D, and the most integrated site did not outperform the others. While FP advice was lower at Clinic D than other sites, FP method provision was similar, and condom provision was much higher (77%).

In total, 32% of sexually active fecund women had unmet FP needs, reported by 46%, 40%, 26% and 25% of women at Clinics A, B, C and D respectively. Other variables crudely associated with unmet FP needs (Table 5) (p<0.05) were having primary or tertiary education, being currently pregnant, parity, having experienced infant death, not having discussed FP with a partner, client type, being pre-ART, and shorter enrolment time in clinic. In the multivariable analysis (also Table 5), there was little evidence that model of care remained associated with unmet FP needs after adjusting for confounding (although some indication that they remained higher at Clinic A compared to D (aOR 2.76, 95%CI 0.88–8.72). Other factors that remained associated with unmet FP needs in the adjusted analysis (p<0.05) were being currently pregnant, being unmarried, parity, having experienced the death of an infant, and not having discussed FP with a partner.

**Table 5:** Crude and adjusted analysis of unmet family planning needs, among sexually active fecund women (n = 286).

			Unmet need					
Variable	Category	N	%	(n)	cOR	95%CI	aOR*	95%CI
Clinic model	Clinic A	(33)	45.5	(15)	2.54	(1.10–5.83)	2.76	(0.88–8.72)
	Clinic B	(86)	39.5	(34)	1.99	(1.05–3.77)	1.19	(0.46–3.07)
	Clinic C	(74)	25.7	(19)	1.05	(0.52–2.12)	0.71	(0.26–1.92)
	Clinic D	(93)	24.7	(23)	1.00		1.00	
Age group	Less than 25	(52)	28.8	(15)	1.03	(0.50–2.13)	2.44	(0.83–7.23)
	25–29	(93)	35.5	(33)	1.40	(0.78–2.51)	1.98	(0.90–4.38)
	30–39	(117)	28.2	(33)	1.00		1.00	
	40 or over	(24)	41.7	(10)	1.82	(0.73–4.50)	1.44	(0.40–5.14)
Marital status	Unmarried	(121)	35.5	(43)	1.34	(0.81–2.22)	2.02*	**(1.03–3.97)**
	Married/living w/ptr	(165)	29.1	(48)	1.00		1.00	
Education	None	(17)	29.4	(5)	1.16	(0.39–3.47)	0.63	(0.16–2.48)
	0–7 yrs (primary)	(70)	41.4	(29)	1.97	(1.11–3.52)	1.76	(0.81–3.83)
	8–12 yrs (secondary)	(182)	26.4	(48)	1.00		1.00	
	> = 12 yrs (college)	(17)	52.9	(9)	3.14	(1.15–8.60)	2.74	(0.70–10.67)
Average monthly income	E<500	(84)	28.6	(24)	0.96	(0.50–1.85)	0.63	(0.25–1.57)
	E500–999	(87)	36.8	(32)	1.40	(0.75–2.62)	1.41	(0.62–3.24)
	E1000–4999	(92)	29.3	(27)	1.00		1.00	
	> = E5000	(23)	34.8	(8)	1.28	(0.49–3.38)	1.25	(0.32–4.93)
Distance from clinic (cost)	E0–E5	(142)	37.3	(53)	1.00		1.00	
	E6–E10	(64)	25.0	(16)	0.56	(0.29–1.08)	0.57	(0.25–1.30)
	E11-E20	(47)	29.8	(14)	0.71	(0.35–1.45)	0.55	(0.22–1.39)
	Over E20	(33)	24.2	(8)	0.54	(0.23–1.28)	0.59	(0.18–1.93)
Current pregnancy	No	(241)	26.1	(63)	1.00		1.00	
	Yes	(45)	62.2	(28)	4.65	(2.39–9.07)	7.31*	**(2.56–20.87)**
No. living children	No children	(44)	15.9	(7)	0.41	(0.17–0.99)	0.14*	(0.04–0.48)
	1–2 children	(149)	31.5	(47)	1.00		1.00	
	3–4 children	(78)	41.0	(32)	1.51	(0.86–2.67)	3.74*	**(1.67–8.37)**
	5 or more children	(15)	33.3	(5)	1.09	(0.35–3.35)	3.62	(0.75–17.57)
Age of youngest child	< = 2 years	(78)	30.8	(24)	0.94	(0.53–1.64)	0.67	(0.30–1.49)
	Over 2 years	(208)	32.2	(67)	1.00		1.00	
Death of child	No	(183)	27.3	(50)	1.00		1.00	
	Yes	(103)	39.8	(41)	1.76	(1.05–2.93)	3.17*	**(1.59–6.31)**
FP discuss with partner	No	(60)	45.0	(27)	2.07	(1.15–3.72)	2.98*	**(1.37–6.48)**
	Yes	(226)	28.3	(64)	1.00		1.00	
Client type	Pre-ART	(31)	38.7	(12)	1.76	(0.80–3.88)	1.75	(0.24–12.70)
	ART initiation	(13)	53.8	(7)	3.24	(1.04–10.11)	1.33	(0.21–8.62)
	ART refill	(189)	26.5	(50)	1.00		1.00	
	ART user consult	(31)	25.8	(8)	0.97	(0.41–2.30)	0.44	(0.14–1.35)
	PMTCT/Infant HIV	(22)	63.6	(14)	4.87	(1.93–12.29)	2.41	(0.36–16.09)
Taking ART	Not on ART	(54)	44.4	(24)	1.97	(1.07–3.62)	0.65	(0.12–3.62)
	On ART	(232)	28.9	(67)	1.00		1.00	
Time enrolled at clinic	<6 months	(88)	40.9	(36)	1.85	(1.05–3.27)	1.02	(0.47–2.23)
	6 months—2 years	(136)	27.2	(37)	1.00		1.00	
	> 2 years	(62)	29.0	(18)	1.09	(0.56–2.13)	1.02	(0.42–2.49)
CD4 count	<50	(17)	41.2	(7)	1.67	(0.60–4.62)	3.51	(0.86–14.33)
	51–200	(80)	33.8	(27)	1.22	(0.69–2.13)	1.40	(0.66–2.98)
	>200	(183)	29.5	(54)	1.00		1.00	
	No count	(6)	50.0	(3)	2.39	(0.47–12.21)	0.99	(0.14–6.93)

*Adjusted for all other variables in table; significant associations (p<0.05) highlighted in bold with *

## Qualitative Findings: Achieving RH-HIV Service Integration in Practice

The quantitative findings described above indicate that integrated sites were not routinely achieving the delivery of RH services to PLHIV, and were no better than a fully stand-alone site at addressing FP needs. We drew on the qualitative data from clients and providers to explore the contextual factors that influenced the provision of care at these four sites. Findings are grouped into individual, interpersonal, infrastructural/systems and institutional factors.

### Individual factors

Provider skills, attitudes and motivation were highly influential. Despite receiving pre-service medical education, most providers considered in-service RH training a pre-requisite to delivering these services as part of HIV care, in particular training on contraceptives. Receiving training was not dependent on integration model, however: providers at the fully stand-alone HIV clinic received in-service training on RH counselling, even though contraceptives were not available on site. Practice on the job was also critical for sustaining skills. Specialisation or partial integration (separate providers delivering different components of RH or HIV) led to a loss of confidence and ‘deskilling’. Formerly generalist providers thus evolved into de-facto ART specialists, relying on other staff with perceived specialist competencies to address differing health needs:

*whenever I can’t properly examine the child*, *I usually call someone from the child welfare department and say “come*, *please let’s examine the child together*, *probably you’ll see something I don’t” [Provider 0102*, *Clinic B*, *partially integrated]*

For providers previously focused on RH, the complexity of ART provision was particularly challenging. At fully integrated Clinic A, providers often internally referred HIV clients to the “ART unit”, to those who they considered more expert in that area. The presence of a new doctor at this clinic also led to nurse discomfort in conducting ART-related tasks, due to fears of criticisms of their skills.

Perceived personal benefits from integrating care were notably absent from provider accounts. While many observed important client benefits (e.g. addressing unmet RH needs, or avoiding problematic referral systems), only one perceived a personal time efficiency benefit. Furthermore, many highlighted potential risks, in particular at stand-alone sites, including putting off men by over-orienting clinics to women’s needs, or diminishing the quality of HIV care. Several feared extended waiting times for clients, which could deter clients, or impact on ART adherence:

*most of the people will be complaining [if I provide RH too] […] they want to be in the queue for two minutes*, *one minute*, *so […] it means I might end up compromising the time I’m supposed to see all the other clients [Provider 0103*, *Clinic B*, *partially integrated]*

While most providers reported job satisfaction from working with HCTx, motivational barriers to integrating care were still discernible, in particular at fully integrated Clinic A. Staff were reportedly reluctant to learn new skills, found HIV provision emotionally challenging, and frequently protested about delivering HIV services; some even resigned due to increased workloads. Consequently, it was reported that nurses would “dump” HIV clients onto the ‘ART providers’ without performing their designated tasks:

*The minute the HIV issue comes up*, *and they test positive*, *yoh*, *(laughs) “go to the ART clinic”—that’s not integration [Provider 0401*, *Clinic A*, *fully integrated]*

As this provider acknowledges, Clinic A was therefore functioning more as a partially integrated clinic, despite all providers having received training on HCTx.

### Interpersonal factors

Provider-client relations were critical. Firstly, a tailored and continuity approach to RH counselling was lacking. FP counselling was almost exclusively focused at treatment initiation, a time when clients reported low sexual activity:

*my [HIV] status has killed my emotional feelings and desire for sex*, *I’m no longer interested in men […] when my partner says we should have sex I feel like climbing on top of the bed and raising an alarm… [Female client 0102*, *Clinic B*, *partially integrated]*

Several providers also agreed that clients did not want to hear RH messaging at this time. While sexual activity often resumed following improvement in health status, RH counselling was usually not repeated. FP counselling also tended to be retrospective, i.e. following unwanted pregnancies, rather than preventive, with documentation of clients’ last menstrual period more common than information on contraceptive use. While documenting pregnancy is essential for HIV care, only one provider acknowledged that “it’s late” for action on FP.

Secondly, subsequent to the intensive adherence counselling received at ART initiation, most clients reported a system where they would move rapidly in and out of ART consultation rooms for check-ups and drug collection. Across all clinics, a routinised model of care was the norm:

*they seem to always be in a rush and so the only thing they ask you is how you’re feeling*, *and then they ask you what you came for today and then they write you a short note to go and take your pills [Female Client 0203*, *Clinic D*, *fully stand-alone]*

Even at Clinic A, where consultation times were measured to be longer and providers espoused the importance of counselling, rushed and ‘task-orientated’ care was still in evidence, reported by both clients and providers:

*[the doctor’s] hurrying of course […] some clients are complaining that he doesn’t even explain to them what he’s doing*, *like he may just give the result and then [say] “go to dispensary”*. *But […] you need to explain what you’ve found […] If you’re just going to be opening the file*, *running through the results*, *not even saying anything about the meaning of those results—you’re not saying anything [Provider 0401*, *Clinic A*, *fully integrated]*

Thirdly, imbalances in power between clients and providers inhibited clients’ articulation of their needs and concerns. Clients were fearful of providers, including their disapproval of FP use or of getting pregnant. They were afraid to ask questions about RH, or for coordinated appointments for different health issues. Such fears could have profound consequences, as this narrative indicates:

*we always educate them not to be pregnant with a low CD4 count*, *so the patient becomes afraid to say that “I’m pregnant now”*, *so you find that they end up abandoning these babies or whatever they do*. *[…] Like last week […] someone decided to put the baby in a pit latrine*. *She was afraid*, *she didn’t tell anyone that she was pregnant […] And when I asked [afterwards]*, *she said “you always ask me if I’m pregnant and I was afraid you were going to say my CD4 [was low]” […] She was just initiated on ART*, *so she knew it was wrong*. *But we don’t scold them… [Provider 0102*, *Clinic B*, *partially integrated]*

In such an account, a complex web of service-related and social influences on health behaviours emerges, including desperation with an unintended pregnancy, deficiencies in health education, social distance and lack of understanding from providers, and potential maltreatment in clinics.

Fourthly, providers failed to address complex client situations and needs. In particular, many had concerns about contraceptive side-effects and pill burdens with ART use. One client, having reported an unintended pregnancy before ART initiation, as well as a desire to use FP, still had not received a method six months later. While she had been advised to attend the clinic’s FP unit, fear of contraceptive side-effects remained a barrier:

*… I want to ask my doctor whether [implants] stay in your body forever*, *maybe he can explain better and maybe see if I can do it*, *but I’m really afraid*. *[…] I haven’t gone [to the FP side] yet… I’m still thinking which method I can use ‘cause when I use the pill I get wet and when I use the injection it hurts when I urinate […] and so I then decided to stop everything [Female client 0102*, *Clinic B*, *partially integrated]*

A referral down the corridor to the FP room was therefore insufficient, and providers had failed to listen to or evaluate her needs and concerns.

Fifthly, the style of counselling was problematic. Clients complained of hearing the same “lectures” each week, which providers themselves acknowledged were ineffective. Some providers, in particular doctors, felt that they lacked training in counselling skills, which might explain shortcomings in this area.

Lastly, inter-professional relationships were also important. Across all sites, while providers relied on colleagues to provide a spectrum of HIV and RH care, inter-professional tensions inhibited a smooth continuum of care. There was frustration when others were not seen to be pulling their weight:

*I’ve tried to decentralize all the duties*, *I’ve really tried to say “I cannot do it alone*, *let’s do it together*, *all of you*, *let’s move*.*” […] Like the issue of pap smears*, *[…] it’s not my baby [sic][job] to come here and do the pap smear when you know that this client is supposed to take a pap smear as a routine [Provider 0401*, *Clinic A*, *fully integrated]*

Expectations of team collaboration in addressing the RH needs of PLHIV were therefore left unfulfilled.

### Infrastructural/systems factors

Space for integrated care was problematic in all facilities. For example, at partially integrated Clinic B where the nurse shared an office with the data clerk, inadequate privacy restricted discussion of personal matters (including RH) and full history-taking. In sites lacking beds, pelvic examinations or IUD insertions were impossible. Privacy was particularly impeded at Clinic C where some ART services were delivered on chairs in the corridor. The fragmentation of care across different providers over time also impacted on RH delivery, which is particularly personal:

*if this person is presenting with an STI*, *then […] I refer*, *I am no more integrating*. *Maybe this person had developed so much […] confidence in me*, *and then I have to say “go”*, *some may not remember the other nurse*.*[…] And besides…*.*telling your story again and again and again*, *it’s not nice*, *is it*? *[Provider 0405*, *Clinic D*, *fully stand-alone]*

The physical co-location of services appeared to facilitate integration success. Locating TB services right next to ART services at the hospital (Clinic C), for example, was reported as a positive development. Conversely, referring clients to distant units was considered problematic by both providers and clients, even if it was only to the other side of the hospital.

Systemic barriers were also critical. Providers across all models of care struggled to address RH with ever-growing numbers of ART clients. Instead, they focused on the immediate task (ART), either telling clients to come back another day, or simply omitting RH counselling:

*it’s not possible to do it under one roof […]*, *like now I’m working alone…I can’t provide ARVs*, *do adherence counselling and compliancy […] and do the immunization in the same room and do family planning*, *you know what I’m saying*? *It’s quite a lot of a job so it’s very impossible*. *[Provider 0104*, *Clinic B*, *partially integrated]*

One provider at Clinic C even reported spending “as little time as possible” with each client, and it was felt that delivering RH was “an overload”. Time pressure was compounded by the complexity of HIV disease management, particularly strenuous around the time of initiation:

*it’s not the only thing that you’re supposed to ask and you’re supposed to do*, *so you may overlook the family planning issue*. *[…] You may not necessarily forget*, *but you may not talk about it*, *because […] a lot of things can come up and you may end up not talking about family planning*, *but would have talked about a lot of other very important things [Provider 0401*, *Clinic A*, *fully integrated]*

Clients were also aware of these pressures, and contributed to shaping patterns of care. They often failed to raise FP or other important health concerns, and those who did also felt guilty about it:

*maybe because I like to ask some questions I think I delay the others*, *I think I take about 15 minutes but the others take less [Male client 0204*, *Clinic D*, *fully stand-alone]*

Individuals delaying the provider was thus seen to result in longer waiting times for all. This situation was also exacerbated by uneven client flow throughout the day and week; most clients would come at the beginning of the day, increasing perceptions of busyness and the need to deliver fast care, while clinics often remained empty and calm towards the end of the day. In Clinic A, the doctor was only onsite two afternoons a week, which led to an influx of HIV clients at those times. As a consequence of perceived time pressures, providers had to prioritise different health concerns, as one indicated, starting “with the bigger issues” first (usually not RH).

Vertical RH and HIV programme structures in the Ministry of Health also had important effects in clinics, including separate data systems, and client registers positioned in different rooms. Some staff were specifically employed for a particular ‘programme’, contributing to de-facto role specialisation. A lack of appropriate guidelines on contraception for PLHIV was also considered problematic, and providers highlighted the need to revise ART registers, forms and booklets to include data on contraception “as a reminder to say “okay, did I talk about this?””.

### Institutional factors

The institutional focus of the clinic also influenced integration. Clinic A, due to its history as a RH clinic, was seen both from within and outside as such, not as an HIV clinic. Consequently, some providers there felt that the managers lacked specialist knowledge and training in HIV medical service delivery. Attracting men to this clinic was also considered challenging. Clients attending other clinics were also not aware that it delivered ART. One female client noted that it would be hard to bring her partner, stating “I think it’s better for him to go where he will be comfortable”. Conversely, one of the reported strengths of fully stand-alone Clinic D was its ability to provide a comfortable environment for men:

*I probably think [men] do feel more comfortable in our setting*, *well men have got issues*, *probably they wouldn’t want to be associated with a unit that is labelled as a sexual reproductive [health] [Provider 0203*, *Clinic D*, *fully stand-alone]*

Clinic fee policies could also inhibit delivery of RH services. While HIV care was ostensibly free everywhere, relatively high fees were charged for RH services at Clinic A (a private NGO clinic, which maintained fees for RH even for PLHIV), and clients there complained about having fees “separated into bits”.

Lastly, while institutional support for integration was in evidence across all sites, this could also be offered in stand-alone sites. Providers at Clinic D reported weekly staff development meetings to build skills in different health areas, including on RH, as well as acting as a forum to address operational problems. The positive managerial ethos evidently made providers happy to work there, with one reporting that they were “the best” ART clinic in Swaziland. Clients were reporting to be “running away” from Clinic C and other locations to attend their clinic, and there was a notable pride in their ability to address wide-ranging client needs, including RH needs, and the needs of the spouses and children of patients.

## Discussion

This comparative case study of four models of care, all serving the same population around one town, has shed light on the realities of integrated care delivery in a high-HIV prevalence setting. By combining quantitative and qualitative methods, it has been possible to both measure whether clients were actually accessing a broad package of RH and HIV services, as well as to explain observed findings through the accounts of providers and clients attending the four sites.

Purported ‘models’ were not always realised in practice. In particular, RH and HIV services were more separated than claimed in ‘fully integrated’ site Clinic A, with internal referral to more specialised HIV providers common. Stand-alone Clinics C and D were able to offer basic RH services, including counselling and condom provision. All clinics demonstrated fragmentation across the discrete tasks of HIV care itself. Generally, receipt of RH services, either on day of survey or since testing HIV positive, was low. This was not driven by low demand, since multiple additional services were still desired, including for RH. Differences in client populations could also explain differential service use patterns, however, including factors such as differences in time since testing between clinics. While not presented here, a multivariable analysis of ‘use of FP services since testing positive’ found those enrolled in the clinic for shorter periods were less likely to have received FP counselling [32]. In this report, we examined unmet FP needs through multivariable methods: while they were found to be crudely higher at integrated sites, logistic regression analysis controlling for socio-demographic, health and RH-related differences in the sample found that there was no strong association with model of care. While more integrated sites are more likely to attract women of reproductive age with a need for contraception, the fact that they were performing no better in addressing FP needs than the fully stand-alone model is surprising, given the greater provision of FP and pregnancy counselling at those sites. One plausible explanation is the content of RH messaging: a linked study has found that FP counselling and FP use in all four models was heavily dominated by condom use [29]. Given that clients struggle to use condoms consistently, this could explain elevated rates of unmet FP needs.

**Fig 1:**
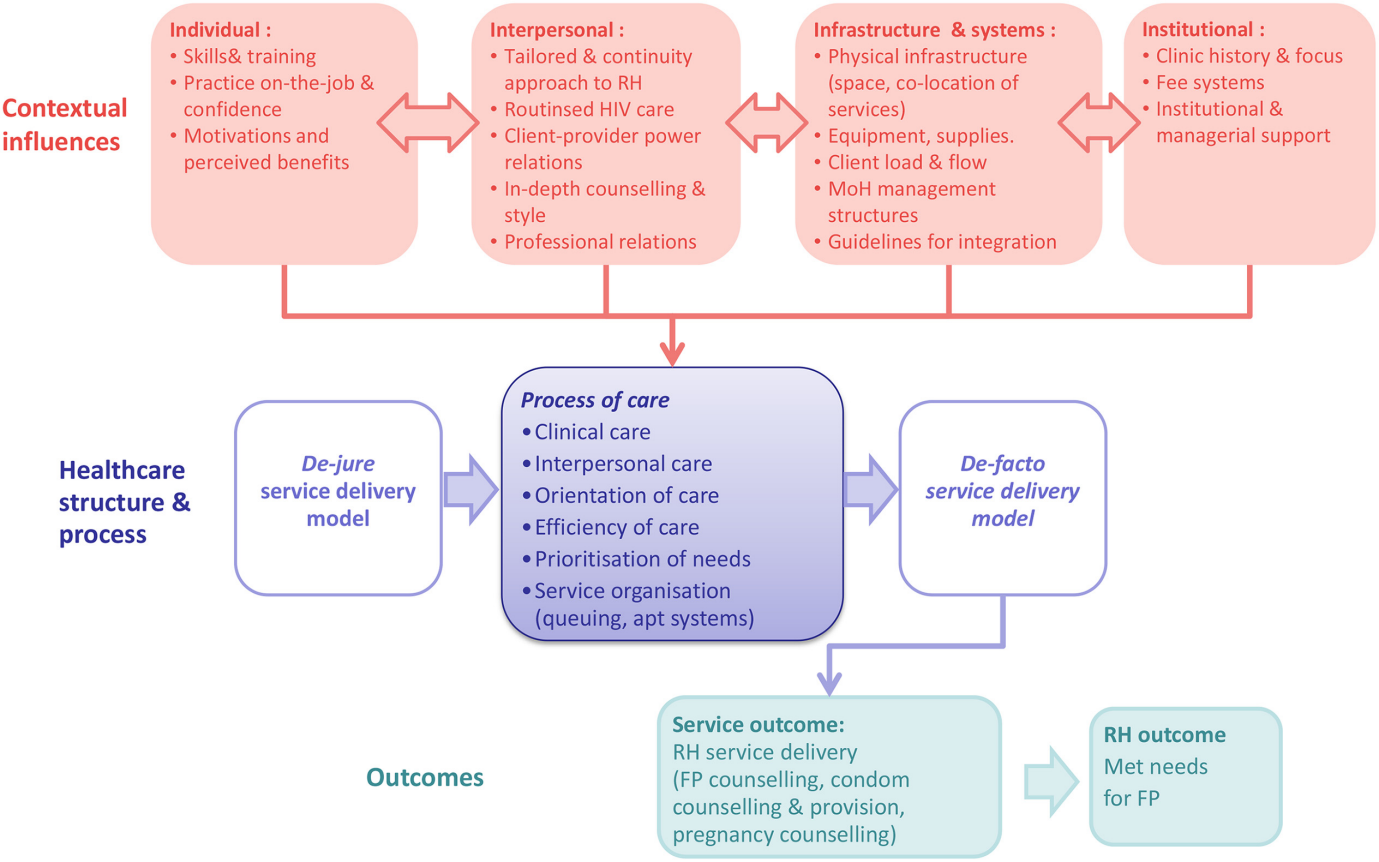
Contextual influences on structure, process and outcomes of integrated RH-HIV care.

Qualitative data suggest that the capacity of clinics to integrate care was also contingent upon a wide range of inter-related social and organisational factors. Fig 1 summarises these contextual factors that influence the process of care, resulting in a de-facto model of service delivery that is different to claimed de-jure models. Many studies on RH-HIV service integration focus primarily on the material resources or systems challenges influencing policy implementation, e.g. staffing, client loads or equipment [18–19], but this study underlines the importance of the social aspects of healthcare, particularly critical client-provider relationships. A linked study demonstrated that clients in these clinics desired personalised and ‘friendly’ care to address multiple health concerns [27], but it was clear that they were often unable to articulate these needs, resulting in drastic consequences for some. Pressurised and rushed care, focused on completion of routine tasks, is clearly limiting, and has been identified as a barrier to integration in other HIV clinics in the region [33]. While all health care invariably involves some degree of routinisation, a more client-centred approach may be required to address the RH needs of PLHIV, with an emphasis on establishing trusting provider-client relationships, shared decision-making, and continuity of care over time [34]. But achieving client-centred care, in turn, may be dependent on integration, suggesting a mutually reinforcing relationship: in-depth counselling is not possible when clients have to visit multiple rooms for sub-components of care. Care continuity was also limited: a shift to providing RH counselling either during pre-ART or in the months following initiation seems warranted, given that the priorities of both providers and clients lay elsewhere at ART initiation.

Overcoming time pressures, influential on both provider and client behaviour, may not be impossible. Care provision was largely in the morning and/or on certain days of the week, indicating additional capacity. Other integration studies have demonstrated similar findings [16], and further research is needed to understand why care is organised this way. The staff workloads described above (‘Study setting’) also demonstrate that clinics with fewer clients were at no advantage. Informal observations in clinics (not reported here), suggest that quieter periods occurred on most days and further research on staff time allocation could investigate whether these periods could be used more productively [32]. While some downtime may be important for administrative purposes, providers’ attempts to blame integration failures on client loads does suggest an externalisation of culpability onto the system, a phenomenon highlighted in sociological studies in other health care settings [35]. A desire to control the system or a lack of trust in clients to cope with appointment systems may also play a role. Moreover, providers’ own motivations and attitudes were clearly influential. Despite most considering themselves ‘generalists’, territorial issues arose where de-facto specialisation occurred, implying a need for greater attention to staff management and policies of team collaboration. They were also inhibited by skills and confidence, and regular practice is clearly required to sustain learned competencies. The ‘deskilling’ of providers through assignment to routine tasks is a challenge that has been infrequently observed or commented on within the health services organisation literature [36]. Given the widespread reliance on team members, it seems that three models could be considered to maintain skills (i) full integration (one provider regularly delivering multiple services); (ii) regular staff rotation; or (iii) opting for semi-specialised services where dedicated providers are trained in and practice specific service sub-components, with well-organised referral mechanisms between different rooms or units. Recent studies have suggested that facilitated referral can be highly effective [37]. Having appropriate tools, registers and guidelines may also help, a finding documented in other studies [38], alongside greater advocacy work with providers to highlight the potential efficiency gains of integration.

The fact that a fully stand-alone clinic was able to achieve positive outcomes also indicates that such models play an important role. Integrated RH-HIV services may be off-putting to men (a key constituent of HIV care), and published findings from other components of this study indicate that client satisfaction is maintained or higher, and HIV-related stigma lower at Clinic D compared to partially integrated models [27–28]. This constitutes a persuasive argument against ‘blanket’ integration policies. A recent trial from Kenya indicates that FP can be successfully integrated into stand-alone HIV clinics, indicating that such models may be a useful policy option [39].

The study has limitations. Generalising findings from four case studies is a concern, but our aim here was to contextualise findings in order to facilitate the transferability of lessons learned in this study to other settings. Regarding quantitative findings, it could be argued that addressing FP needs is not wholly dependent on the actions of the client’s current facility. However, only 15% of clients had previously switched clinics. A sensitivity analysis was conducted excluding those who had accessed services elsewhere, and found no important effect on estimates (all ≤10%) in the multivariable model. Recall bias on services received was likely, but should have been consistent across sites. Disentangling causal mechanisms within the multivariable model was also problematic. Current pregnancy could be construed as an outcome, rather than determinant, of unmet need. However, unmet need is measured in different ways among pregnant women, and given large differences in current pregnancy prevalence between sites, it was critical to retain this variable in the model. Those who tested positive during a current pregnancy were excluded from the analysis, however. And while the study was powered to detect differences in unmet FP needs, a smaller sample size achieved than planned at Clinic A may have contributed to the weak evidence of effect there. A modelling approach using many co-variates may have exacerbated this problem, since such models can be underpowered [40].

The qualitative component also had limitations. Supplementing interview data with observations of care and interviews with clinic managers could have been useful. Ethnographic studies into the workings of HIV clinics have been useful in South Africa for exploring the organisation of care in greater depth [14]. Nonetheless, the triangulation of client and provider interview data helped overcome this limitation. There were also limitations to the scope of the data collection and analysis. Only two doctor-managers and one nurse-manager were interviewed (at Clinics C and D), and it would have been useful to have had a managerial perspective across all sites. Provider data, in particular, may have been heavily influenced by the interaction with the interviewer, a white British female, and particularly positive views of the concept of integration could have been given due to the nature of the research topic.

## Conclusions

This study contributes to the body of evidence indicating that achieving service integration is highly challenging in practice. It highlights the range of contextual factors influencing the capacity to integrate care, and emphasises the social aspects of provider behaviour and client-provider interactions. While HIV clinics continue to be pressurised by heavy client loads in high HIV prevalence contexts in sub-Saharan Africa, mechanisms need to be found to ensure that RH needs are not neglected in the prioritisation process. Norms of task-oriented routinised HIV care must be overcome if integration is to achieve its intended aims of addressing multiple health needs. Findings demonstrate that a well-run stand-alone clinic with institutional support for RH goals can achieve positive outcomes, even without providing contraceptive supplies on site. Integration of HIV into existing RH settings also needs careful management to ensure that de-facto partial specialisation is not maintained or created.

## Supporting Information

S1 Fig Conceptual model for multivariable analyses. (DOCX)

S1 Table Individual component services received on day of survey. (DOCX)
