# Supplementary figures and images for: Exploring the Feasibility of Service Integration in a Low-Income Setting: A Mixed Methods Investigation into Different Models of Reproductive Health and HIV Care in Swaziland
Source: PLoS One. 2015 May 15;10(5):e0126144. doi: 10.1371/journal.pone.0126144 (PMC4433110; doi:10.1371/journal.pone.0126144)

**SUPPLEMENTARY FIGURES AND TABLES**

**Figure S1: Conceptual model for multivariable analyses**

**
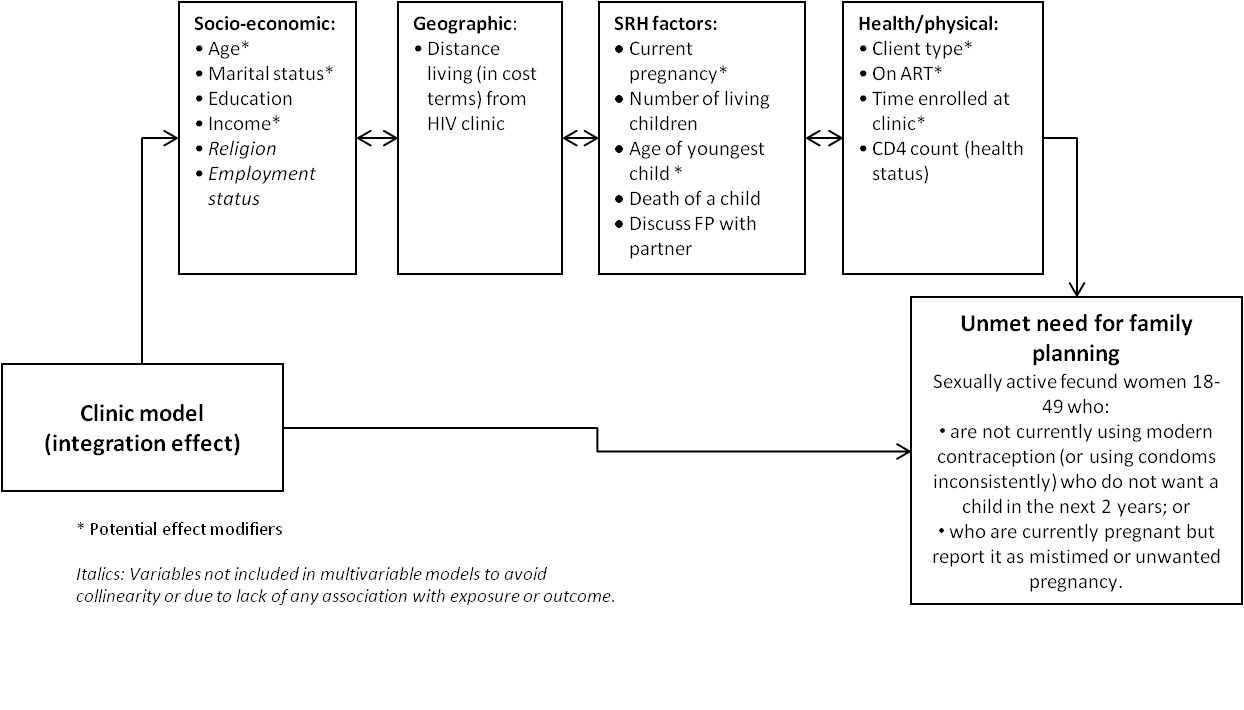
**

Supplement: S1 Fig — (DOCX) [file pone.0126144.s001.docx]
