# Supplementary material for: Exploring the Feasibility of Service Integration in a Low-Income Setting: A Mixed Methods Investigation into Different Models of Reproductive Health and HIV Care in Swaziland
Source: PLoS One. 2015 May 15;10(5):e0126144. doi: 10.1371/journal.pone.0126144 (PMC4433110; doi:10.1371/journal.pone.0126144)
Supplement: S1 Table — (DOCX) [file pone.0126144.s002.docx]

**SUPPLEMENTARY FIGURES AND TABLES**

**Table S1: Individual component services received on day of survey**

|  | **Clinic A** | | **Clinic B** | | **Clinic C** | | **Clinic D** | | **All clinics** | |
| --- | --- | --- | --- | --- | --- | --- | --- | --- | --- | --- |
|  | **(Fully integrated)** | | **(Partially integrated)** | | **(Partially stand-alone)** | | **(Fully stand-alone)** | |  |  |
| **HIV and RH services accessed on day of survey (% of services received , (N))** | | | | | | |  |  |  |  |
| General HIV consult | 26.4 | (56) | 10.9 | (41) | 6.0 | (33) | 8.2 | (42) | 10.4 | (172) |
| Weighing/BP check | 16.5 | (35) | 13.1 | (49) | 29.1 | (161) | 15.9 | (82) | 19.7 | (327) |
| Exams/tests | 8.5 | (18) | 11.5 | (43) | 5.2 | (29) | 5.6 | (29) | 7.2 | (119) |
| ART refill | 9.4 | (20) | 10.1 | (38) | 25.1 | (139) | 30.1 | (155) | 21.3 | (352) |
| ART initiation | 5.7 | (12) | 1.1 | (4) | 0.9 | (5) | 0.4 | (2) | 1.4 | (23) |
| Pre ART consult | 6.1 | (13) | 4.8 | (18) | 2.0 | (11) | 3.3 | (17) | 3.6 | (59) |
| Drug provision | 19.8 | (42) | 31.2 | (117) | 27.8 | (154) | 36.5 | (188) | 30.3 | (501) |
| Group counselling | 0.5 | (1) | 1.9 | (7) | 4.0 | (22) | 0.0 | (0) | 1.8 | (30) |
| Condom provision | 0.9 | (2) | 0.3 | (1) | 0.0 | (0) | 0.0 | (0) | 0.2 | (3) |
| FP | 0.9 | (2) | 0.3 | (1) | 0.0 | (0) | 0.0 | (0) | 0.2 | (3) |
| ANC/PNC/infant | 1.9 | (4) | 10.7 | (40) | 0.0 | (0) | 0.0 | (0) | 2.7 | (44) |
| Pap smear | 0.9 | (2) | 0.0 | (0) | 0.0 | (0) | 0.0 | (0) | 0.1 | (2) |
| PMTCT | 1.4 | (3) | 3.2 | (12) | 0.0 | (0) | 0.0 | (0) | 0.9 | (15) |
| STI | 0.9 | (2) | 1.1 | (4) | 0.0 | (0) | 0.0 | (0) | 0.4 | (6) |
| **Total no. svcs** | **100.0** | **(212)** | **100.0** | **(375)** | **100.0** | **(554)** | **100.0** | **(515)** | **100.0** | **(1656)** |
